# Books and Pamphlets Received

**Published:** 1893-03

**Authors:** 


### BOOKS AND PAMPHLETS RECEIVED.

"The Hydriatic Treatment of Typhoid Fever." by Chr. Sihrler, M. D., Ph. D. Published by Chr. Sihrler.

"The Physician; His Relation to the Law, and Legal Rules Governing the Collection of His Fees." By H. G. Blaine, A. M., M. D. Blaine Bros., Publishers.

"Ague and Alopecia." By L. Duncan Bulkley, M. D. Geo. S. Davis, Detroit, Publisher.

"Cerebral Meningitis." By Martin W. Barr, M. D. Published by Geo. S. Davis. Detroit.

"Fissure of the Anus and Fistula in Ano." By Dr. Lewis H. Adler, Jr. Geo. S. Davis, Detroit, Publisher.

Medical Society of New Jersey. Transactions, 1892.

Report of the Surgeon-General of the Army to the Secretary of War, for the fiscal year ending June 30, 1892.

Transactions of the Medical Society of Virginia, 1892.

"A System of Genito Urinary Diseases, Syphilology and Dermatology, Vol. I." By Prince A. Morrow, A. M., M. D. Published by D. Appleton & Co.

"International Clinics—Vol. IV. Second Series." Published by J. B. Lippincott Co.

"Mental Diseases." By H. P. Stearns, A. M., M. D. Published by Blakiston, Son & Co.

"Piperajin in the Treatment of Stone in the Kidney; Report of Cases." By D. D. Stewart, M. D., Philadelphia, Pa.

"Dudleys Operation on the Cervix, with Report of Five Cases." By. T. D. Marion, M. D., Chester S. C.

"Abscess Around the Rectum." By Charles B. Kelsey, M. D., New York.

"Remarks on So-called Urethral Fever." By G. Frank Gydston, M. D. Chicago.

